# Novel diffusive gradients in thin films technique to assess labile sulfate in soil

**DOI:** 10.1007/s00216-016-9801-8

**Published:** 2016-08-04

**Authors:** Ondrej Hanousek, Sean Mason, Jakob Santner, Md Mobaroqul Ahsan Chowdhury, Torsten W. Berger, Thomas Prohaska

**Affiliations:** 1 Department of Chemistry - VIRIS Laboratory, University of Natural Resources and Life Sciences Vienna, Konrad-Lorenz-Strasse 24, 3430 Tulln, Austria; 2 Institute of Forest Ecology, University of Natural Resources and Life Sciences Vienna, Peter-Jordan-Strasse 82, 1190 Vienna, Austria; 3 School of Agriculture, Food and Wine, University of Adelaide and the Waite Research Institute, Adelaide, South Australia 5064 Australia; 4 Division of Agronomy, University of Natural Resources and Life Sciences Vienna, Konrad-Lorenz-Strasse 24, 3430 Tulln, Austria

**Keywords:** Diffusive gradients in thin films (DGT), ICP-MS, Sulfur, Sulfate, Soil

## Abstract

A novel diffusive gradients in thin films (DGT) technique for sampling labile soil sulfate was developed, based on a strong basic anion exchange resin (Amberlite IRA-400) for sulfate immobilization on the binding gel. For reducing the sulfate background on the resin gels, photopolymerization was applied instead of ammonium persulfate-induced polymerization. Agarose cross-linked polyacrylamide (APA) hydrogels were used as diffusive layer. The sulfate diffusion coefficient in APA gel was determined as 9.83 × 10^−6^ ± 0.35 × 10^-6^ cm^2^ s^−1^ at 25 °C. The accumulated sulfate was eluted in 1 mol L^−1^ HNO_3_ with a recovery of 90.9 ± 1.6 %. The developed method was tested against two standard extraction methods for soil sulfate measurement. The obtained low correlation coefficients indicate that DGT and conventional soil test methods assess differential soil sulfate pools, rendering DGT a potentially important tool for measuring labile soil sulfate.

## Introduction

Sulfur (S) is a plant macronutrient as part of e.g. amino acids, proteins, and coenzymes. It is involved in the plant metabolism as well as in the response to oxidative stress [1]. Sulfur deficiency in arable soils has been reported to become one of the major limitations in crop production [2]. Plants take up S as sulfate (SO_4_^2−^) from the soil porewater. Therefore, determination of labile soil SO_4_^2−^ is essential for the investigation of S phytoavailability in soils [1–3].

Batch extraction techniques using different extractant solutions, e.g., H_2_O, 0.03 mol L^−1^ KH_2_PO_4_, and 1 mol L^−1^ HCl [4, 5], are the most common methods to assess readily available, adsorbed, and carbonate-occluded soil sulfate, respectively. Common agricultural S testing methods include the KCl-40 test, which uses 0.25 mol L^−1^ KCl as an extractant [6]. This method was proposed to be more representative for plant-available soil S than the MCP-S method (using 0.01 mol L^−1^ Ca(H_2_PO_4_)_2_), as KCl-40 provides a measure of adsorbed and soluble SO_4_^2−^, including gypsum.

Tension lysimeters or suction cups are alternative methods for assessing dissolved soil S by directly taking soil porewater samples [1, 3]. However, they do not account for the reversibly adsorbed fraction of soil SO_4_^2−^. Sampling strategies employing ion resins as SO_4_^2−^ sinks, which deplete SO_4_^2−^ in the soil porewater and thereby induce desorption from the solid phase, have been developed to additionally account for this soil SO_4_^2−^ pool [7]. This approach has the additional advantage of pre-concentrating SO_4_^2−^ on the resin. However, if the resin is directly exposed to the soil [7, 8], it may be easily contaminated with soil particles [8]. Competition of other anions (e.g., phosphate, nitrate) for binding sites of the resin may also lead to sampling artefacts [9].

Diffusive gradients in thin films (DGT) is an advanced sink technique, in which the resin is embedded in a hydrogel layer and is covered by a pure hydrogel disc and a protective membrane. This setup prevents particle contamination effectively and allows for the calculation of the time-averaged analyte (SO_4_^2−^) concentration due to the well-defined diffusion geometry [10]:

1 $$ {c}_{\mathrm{DGT}}=\frac{M\cdot \Delta g}{D\cdot A\cdot t} $$

*M* is the mass of analyte bound on the resin layer, Δ*g* is the diffusive layer thickness, *D* is the diffusion coefficient of the analyte in the diffusive layer, *A* is the sampling area, and *t* is the sampling time.

The DGT methodology has been shown to perform exceedingly well in assessing the bioavailable solute fraction if the solute availability is limited by diffusion [11]. Several studies demonstrated that soil phosphate assessed by DGT correlated better with plant phosphate uptake [11–13] and with crop yield responses to applied P [14] compared to conventional batch extractions or other resin-based sampling techniques [11, 13]. Guppy and Blair [15] have shown that established methods (KCl-40 and MCP) were poor at predicting maize S uptake and responses to S applications in a short-term glasshouse experiment. Therefore, an improved, simple, and quick laboratory method like DGT for determining available S could have significant benefits.

No DGT method for SO_4_^2−^ sampling is currently available. The only S species for which a DGT method is available is sulfide, which is sampled by the conversion of AgI to Ag_2_S [16]. As sulfate sorption to oxide minerals (e.g., ferrihydrite, zirconium oxide) [17, 18], which have been used for measuring oxyanions (e.g., PO_4_^3−^and AsO_4_^3−^) by DGT so far, is weak, a general anion exchange resin is the material of choice for sampling sulfate with DGT.

In this study, we present a novel DGT technique for the sampling of labile soil SO_4_^2−^. The developed anion exchange resin gel was characterized (regarding its SO_4_^2−^ uptake capacity, pH working range, and elution efficiency) for applications in soil. Comparison of its performance with traditional techniques assessing soil SO_4_^2−^, the KCl-40, and MCP extractions, shows that DGT samples a different SO_4_^2−^ pool and is therefore a potential alternative for soil SO_4_^2−^ testing.

## Materials and methods

### General laboratory procedures

All consumables were double acid washed using 10 % (*w*/*w*) and 1 % (*w*/*w*) HNO_3_ (p. a., Merck, Darmstadt, DE) and rinsed with laboratory water type I (0.055 μS cm^−1^; TKA-GenPure, Niederelbert, DE) before use. Laboratory water type I was used for preparation of all standard solutions, for soil extractions, and for water saturation of soil samples. Laboratory water type I and HNO_3_ were further purified by a sub-boiling distillation system (Milestone Inc., Shelton, CT, USA) and used for the elution of SO_4_^2−^ from the resin gel (1 mol L^−1^ HNO_3_) and for microwave-assisted digestions (Multiwave 3000, Anton Paar, Graz, AT).

### Diffusive and resin gel preparation

Agarose cross-linked polyacrylamide (APA) diffusive hydrogels of 0.8 mm thickness were prepared according to [10] and cut to discs. Amberlite IRA-400 (chloride form, Sigma-Aldrich, Buchs, CH) resin was selected as a binding agent for SO_4_^2−^. The resin was ground with a ball mill for 10 min, passed through a 200-μm sieve, and washed in 10 % HCl (p.a., Merck), repeating this step twice followed by four rinses with pure water, to reduce the background S on the resin.

The common acrylamide polymerization technique used for DGT gels applies ammonium persulfate (APS) as initiator [10]. This approach is not suitable for preparing resin gels for the sampling of SO_4_^2−^, as elevated background S levels on the binding gel can be expected. To reduce background S levels, photopolymerization using riboflavin ((−)-riboflavin, Sigma-Aldrich) as photoinitator was applied. The polymerization was started through the decomposition of riboflavin upon exposition to a light source. A detailed study on the riboflavin-initiated polymerization of acrylamide was published e.g. by Oster et al. [19].

Three grams (wet weight) of ground and washed Amberlite IRA-400 were mixed with 10 mL gel solution prepared as described in [10] and 60 μL of riboflavin solution (0.01 g riboflavin in 10 mL H_2_O) and 20 μL of tetramethylethylendiamine (TEMED; VWR Int., Randor, USA) were added. The solution was shaken well and cast between two acid-washed glass plates (6 × 20 cm) separated by a U-shaped acid-washed plastic spacer (0.4 mm thickness). The glass plate with the gel solution was left under fluorescent light overnight. Gels appeared to set after about 1 h. The resin gels produced in this way were relatively weak and subject to tearing. While avoiding the binding of SO_4_^2−^ from APS to the resin gels was important for preventing elevated S background levels, such precautions are not necessary for diffusive gels, which have no capability for SO_4_^2−^ binding. Therefore the diffusive gels used in this study were produced using the classical procedure [10]. Any residual S introduced as APS was washed off the diffusive gels during the gel hydration step. A 10 mmol L^−1^ NaNO_3_ solution was used for storage of all gels (Reagent Plus, Sigma-Aldrich).

**Fig. 1:**
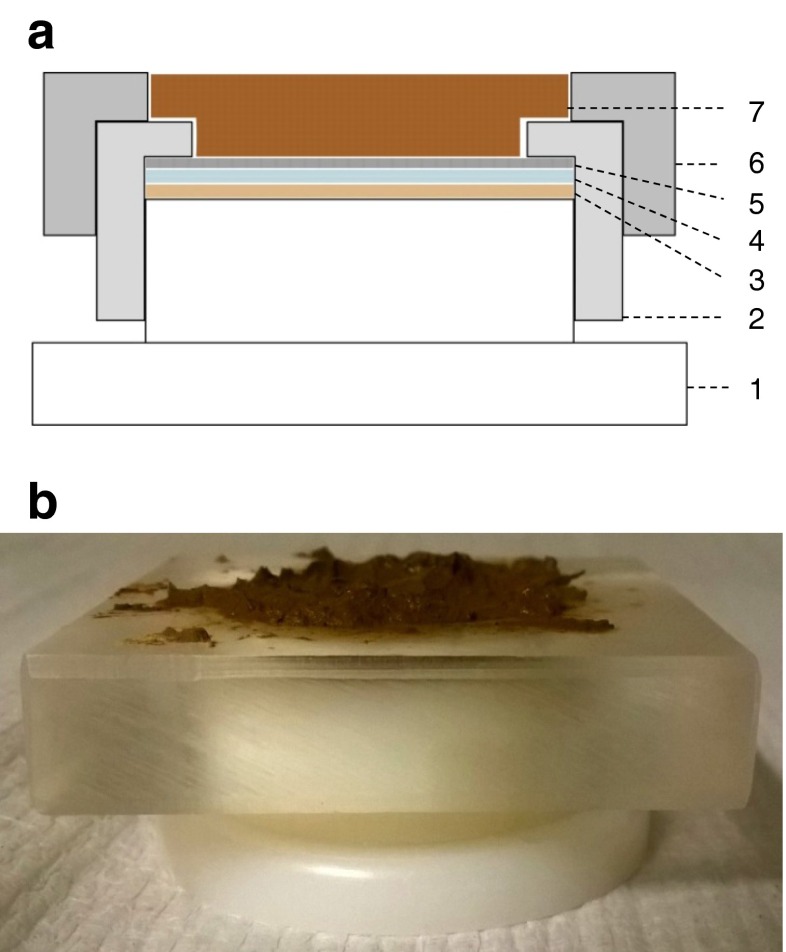
DGT sampling device schematic (**a**): *1* piston, *2* outer sleeve with sampling window, *3* resin (Amberlite IRA-400) gel, *4* diffusive (APA) gel, *5* protective membrane, *6* plastic frame to hold the soil sample in place, *7* soil sample. Application of the DGT device to soil (**b**)

Polyethersulfone filters (0.45 μm pore size, 0.13 mm thick, Sartorius Stedim, Goettingen, DE) were used as a protective membrane. The filters were washed with 1 mol L^−1^ HNO_3_ overnight and stored in 10 mmol L^−1^ NaNO_3_. DGT samplers (DGT Research Ltd., Lancaster, UK) were used for both solution and soil tests. A schematic of the DGT device is pictured in Fig. 1a. Figure 1b shows the application to soil (see “Comparison of DGT S with conventional soil S extraction techniques” section).

### Evaluation of DGT sampling

#### Sulfur background level

The sulfur contents in the protective membrane (acid-washed and unwashed), in the diffusive gel, and in the resin gel (S background concentrations) were determined after elution in 10 mL 1 mol L^−1^ HNO_3_ for 16 h and calculated as S amount per membrane or gel disc. The S content of the eluent was used to determine the instrument limit of detection of the inductively coupled plasma mass spectrometer (ICP-MS, see “ICP-MS” section) (limit of detection (LOD) = average eluent S content + 3 × standard deviation).

The method blank (resin gel, diffusive gel, and protective membrane in DGT sampler placed for 4 h in a moist plastic bag at 21 °C) was measured in 10 mL 1 mol L^−1^ HNO_3_ eluate of the resin gel and calculated as S amount per resin gel disc. The S contents were used to determine the method limit of detection for S by ICP-MS (method limit of detection (MDL) = average method blank S content + 3 × standard deviation).

#### Resin gel elution efficiency (*R*)

A recovery experiment using a ^35^S radiotracer (Perkin Elmer, Waltham, MA, USA) was conducted. 10 mL 0.5 mg L^−1^ S ((NH_4_)_2_SO_4_, p.a., Merck) and 10 mL 10 mg L^−1^ S ((NH_4_)_2_SO_4_) solutions were spiked with ~980 Bq ^35^S. A resin gel was immersed in each solution. The solutions were shaken for 4 h and the gels were eluted in 10 mL 1 mol L^−1^ HNO_3_ for 16 h subsequently. This experiment was repeated five times.

#### Diffusion coefficient (*D*)

The diffusion coefficient *D* of SO_4_^2−^ in APA gel was determined using a diffusion cell [20]. The cell consisted of two 110 mL Perspex containers, each with a 1.59-cm-diameter opening. A 2.5-cm-diameter diffusive gel disc was placed between the openings and the containers were clamped together. One hundred milliliters of water (pH 5.6) were introduced into one of the containers and the tightness of the clamping was checked. Then, 100 mL (NH_4_)_2_SO_4_ (p.a., Merck) solution (pH 5.6) was introduced into the second container. To ensure that *D* is not concentration dependent, sulfate solutions of 1, 10, and 45 mg L^−1^ S were used. Each sulfate solution was spiked with ~370 k Bq ^35^S. Solutions in both containers were stirred continuously. Subsamples were taken from both containers in time intervals of about 30 min to follow the diffusion of S through the APA gel. *D* and its uncertainty were calculated as described in [21]. The final coefficient *D*_25_ was calculated as a mean value of the diffusion coefficients determined in the 1, 10, and 45 mg L^−1^ S solutions at 25 °C. The *D* value in further experiments was calculated from *D*_25_ by temperature adjustment using Eq. 2 [10]:

2 $$ \log {D}_t=\frac{1.37\left(t-25\right)+8.36\times {10}^{-4}{\left(t-25\right)}^{-2}}{109+t}+ \log \frac{D_{25}\left(273+t\right)}{298} $$

#### pH working range

Sulfate as (NH_4_)_2_SO_4_ was dissolved in laboratory water type I to reach a S concentration of 4–5 mg L^−1^. The solutions (3 L each) were stirred until equilibrium with air was reached and pH was stable. The pH of the solutions was set to 2.98, 3.48, 3.55, 3.97, 4.00, 5.00, 5.10, 5.60, 6.12, 7.02, 7.40, 8.18, 8.34, and 9.05 using dilute HNO_3_ and NaOH. Three to four DGT samplers were exposed to each solution for 4 h. The temperature was monitored throughout the experiments. The calculated *c*_DGT_ values (Eq. 1) were compared to the S concentration in the corresponding immersion solution, *c*_soln_.

#### Gel capacity

A synthetic soil solution was prepared for testing the resin gel capacity for SO_4_^2−^ uptake under realistic conditions, i.e., taking the competing anion species chloride, nitrate, and phosphate into account. The concentration of SO_4_^2−^ was chosen based on typical porewater SO_4_^2−^ concentrations [22]. For obtaining a realistic and conservative estimate of the gel SO_4_^2−^ capacity, concentrations of Cl^−^, NO_3_^−^, and PO_4_^3−^, based on upper level of the concentration ranges of own and literature soil solution data [3, 22], were chosen. Sulfate as (NH_4_)_2_SO_4_ was dissolved in laboratory water type I (6 L) to reach a SO_4_^2−^ concentration of 15 mg L^−1^. NaCl, NaNO_3_, and KH_2_PO_4_ were added to the SO_4_^2−^ containing solution to reach concentrations of 9.0, 60, and 7.5 mg L^−1^ of Cl^−^, NO_3_^−^, and PO_4_^3−^, respectively. DGT samplers (21 in total) were placed into this solution. After 3, 6, 9, 15, 24, 39, and 48 h, three samplers were taken out at a time. Temperature and pH were monitored during the experiment. The resin gels were eluted in 1 mol L^−1^ HNO_3_ subsequently. The content of SO_4_^2−^, Cl^−^, and PO_4_^3−^ in the eluates was measured (NO_3_^−^ content could not be measured as HNO_3_ was used for elution). The gel capacity was estimated as the highest mass accumulated on the gel that did not differ significantly from the theoretical mass uptake according to Eq. 1.

### Analyses

#### ICP-MS

A single collector sector field ICP-MS (Element XR, Thermo Fisher Scientific, Waltham, MA, USA) was used for S quantification in standard solutions and resin gel eluates during method development in the VIRIS Laboratory, Tulln (AT). External calibration (0–3 mg S L^−1^) and internal standardization (using 1 μg L^−1^ In) were applied. The instrumental LOD was 3 μg S L^−1^.

#### ^35^S radiotracer

Isotope dilution using ^35^S was used when very low S concentrations were expected (determination of diffusion coefficient) and for validation of some data obtained by ICP-MS measurement (elution efficiency). A liquid scintillation counter Tri-Carb 2910 TR (PerkinElmer) was used for measuring the beta radiation emitted by ^35^S. Measurements were performed on a comparative basis, i.e., the activity of the eluates was compared to the activity of immersion solutions for the determination of the elution efficiency. The gradual increase of activity in subsamples in time was used for determination of the diffusion coefficient.

#### ICP-OES

All DGT eluents and extraction solutions from the soil survey (see “Comparison of DGT S with conventional soil S extraction techniques” section) were measured for their S content using ICP-OES (Optima 7000 DV, PerkinElmer) at 181.975 nm at the University Adelaide (AU). External calibration (0–10 mg S L^−1^) was applied. The instrumental limit of detection was 20 μg S L^−1^.

### Uncertainty estimation

The calculation of the combined uncertainty of the elution efficiency (*u*_R_, Eq. 3) was based on the combination of the measurement repeatability (SD_1_, *n* = 5) and reproducibility (SD_2_, *n* = 3), combining thus both sample heterogeneity and measurement reproducibility:

3 $$ {u}_R=\sqrt{{\mathrm{SD}}_1^2+{\mathrm{SD}}_2^2} $$

The uncertainty of the quantitative measurement was calculated by applying the approach of partial derivatives (*u*_*c*DGT_, Eq. 4) based on [21]. Its calculation included the uncertainties of S quantification (*u*_MEAS_, which comprises measurement precision of analyte and of internal standard, blank correction uncertainty, and uncertainty of calibration), the uncertainty of the diffusive layer thickness (*u*_DL_), the uncertainty of the sampling window surface area (*u*_A_) and the uncertainty of the diffusion coefficient (*u*_D_, comprising the uncertainty of the slope of the mass vs. time line, of the thickness of the diffusive gel, of the surface area of the connection of the diffusion cell halves, and of the original concentration of S in the solution), the sampling time (*u*_t_), and the elution efficiency (*u*_R_):

4 $$ \frac{u_{c\mathrm{D}\mathrm{G}\mathrm{T}}}{c_{\mathrm{DGT}}}=\sqrt{{\left(\frac{u_{\mathrm{MEAS}}}{c_S}\right)}^2+{\left(\frac{u_{\mathrm{DL}}}{\mathrm{DL}}\right)}^2+{\left(\frac{u_A}{A}\right)}^2+{\left(\frac{u_D}{D}\right)}^2+{\left(\frac{u_t}{t}\right)}^2+{\left(\frac{u_R}{R}\right)}^2} $$

where *c*_S_ is the determined S concentration in the eluate, DL is the diffusive layer thickness, *A* is the sampling window area, *D* is the diffusion coefficient, *t* is the sampling time, and *R* is the elution efficiency.

Estimation of the uncertainty of the *c*_DGT_/*c*_soln_ ratio, which was e.g. used for determining the pH working range, comprised both the uncertainty of the *c*_DGT_ value (*u*_*c*DGT_, Eq. 4) and the uncertainty of the determination of the S concentration in the immersion solution (*u*_MEAS2_, which comprises measurement precision of analyte and of internal standard, blank correction uncertainty, and uncertainty of calibration).

5 $$ \frac{u\left({c}_{\mathrm{DGT}}/{c}_{\mathrm{soln}}\right)}{c_{\mathrm{DGT}}/{c}_{\mathrm{soln}}}=\sqrt{{\left(\frac{u_{c\mathrm{D}\mathrm{G}\mathrm{T}}}{c_{\mathrm{DGT}}}\right)}^2+{\left(\frac{u_{\mathrm{MEAS}2}}{c_{\mathrm{soln}}}\right)}^2} $$

Significance of a difference between mean values (*c*_DGT_/*c*_Soln_ vs. 1.0 line in pH working range determination and experimental vs. theoretical DGT uptake in gel capacity determination) was tested with respect to the expanded uncertainties (*U* = 2 × *u*_*X*_) of the mean values to cover 95 % confidence interval. Two mean values were significantly different, if

6 $$ \left|{m}_1-{m}_2\right| > \sqrt{U_{m1}^2+{U}_{m2}^2} $$

where *m*_1_ and *m*_2_ represent the mean values and *U*_m1_ and *U*_m2_ their expanded uncertainties [23].

### Comparison of DGT S with conventional soil S extraction techniques

We assessed the relation of DGT sampled S and S extracted by two conventional soil extraction methods (KCl-40, MCP) of eight agricultural soils (see Table 1) from major cropping regions in Australia. The soil parameters were determined by standard methods following [24]. To determine S by KCl-40, 4.5 g of air-dried soil were extracted in 30 mL 0.25 mol L^−1^ KCl at 40 °C. The mixture was incubated for 3 h at 40 °C and was repeatedly shaken by hand. The supernatant was separated by centrifugation. To determine MCP-S, 20 g of air-dried soil was mixed with 100 mL 0.01 mol L^−1^ Ca(H_2_PO_4_)_2_ at pH 4. The mixture was shaken over-head for 17 h. The supernatant was separated by centrifugation [24]. For the DGT technique, soils were moistened to 100 % water holding capacity (WHC) 1 day prior to deployment. Six DGT devices were deployed on each soil for 6 h at a constant temperature of 21 °C (see Fig. 1b). After deployment, DGT devices were rinsed with laboratory water type I and the binding gels were retrieved and eluted.

**Table 1:** Soil properties

Soil name	Abbreviation	State of origin (Australia)	Texture	Clay	WHC	pH	*C*_org_	*N*_tot_
				%	%	(CaCl_2_)	%	%
Birchip	BI	VIC	Medium clay	31	24.9	7.7	0.75	0.13
Hart	HA	SA	Medium clay	38	52.8	6.4	1.49	0.16
Karoonda	KA	SA	Sand	2	20.1	5.4	0.39	0.08
Keith	KE	SA	Sandy clay	15	20.2	5.0	1.94	0.17
Lake Bolac	LB	VIC	Sand	3	39.4	5.9	1.33	0.15
Mt Barker	MB	WA	Sand	12	28.7	5.6	2.49	0.20
Otterbourne	OT	ACT	Medium clay	13	32.5	5.4	3.00	–
Tumby Bay	TB	SA	Sandy clay	17	22.4	4.6	3.00	0.23

*WHC* water holding capacity

## Results

### Blank levels, LOD, diffusion coefficient, elution efficiency

**Fig. 2:**
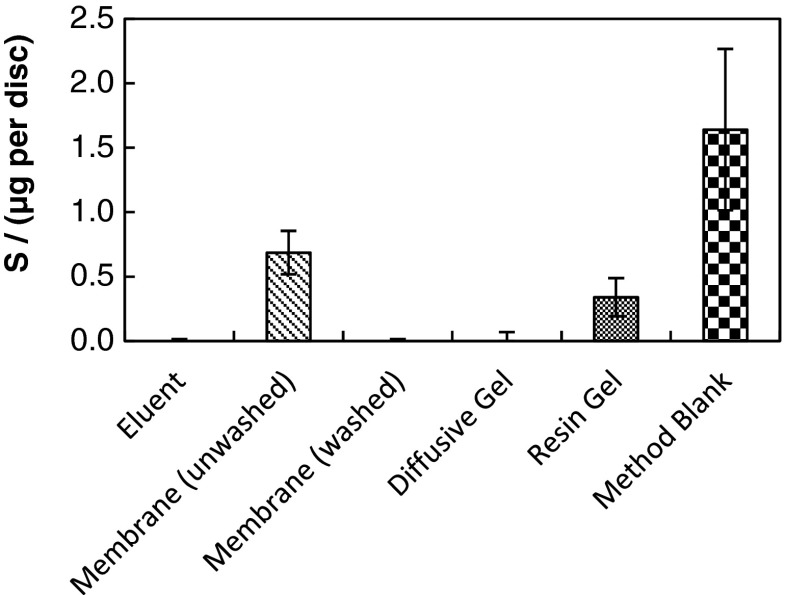
Background signal of the eluent (1 mol L^−1^ HNO_3_) and S loadings of eluted membranes and gels. Washed membrane and diffusive gel are below instrument LOD. *Error bars* are 1 SD (*n* = 4)

The background S signals measured in the eluent (1 mol L^−1^ HNO_3_), in eluates of the acid-washed membrane, and of the diffusive gel were below the instrument limit of detection of ICP-MS (see Fig. 2). The background S content of the unwashed protective membrane and resin gel reached 0.69 ± 0.17 μg S per membrane disc and 0.34 ± 0.15 μg S per gel disc (average ± 1 SD), respectively.

The MDL was 0.29 mg S L^−1^. The S loading of the resin gel in the method blank was 1.64 ± 0.63 μg S per gel disc (average ± 1 SD). For some gel discs, the loading reached up to 2.23 μg S. However, some batches of the resin gel showed S loadings below the instrument LOD.

**Fig. 3:**
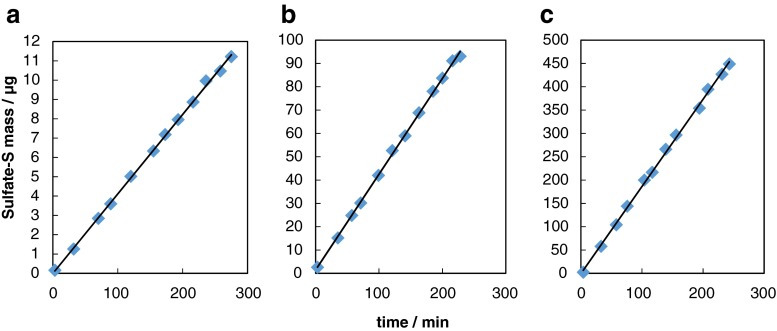
Mass transport of sulfate-S through the APA diffusive gel over time. Using the slope of the regression line and the initial S concentration (**a** 1 mg L^−1^, **b** 10 mg L^−1^, **c** 45 mg L^−1^), *D* was calculated according to [20]

The mass S diffused through the APA diffusive gel over time is displayed in Fig. 3. The diffusion coefficient of SO_4_^2−^ at 25 °C (*D*_25_) calculated from these slopes was 9.83 × 10^−6^ ± 0.35 × 10^−6^ cm^2^ s^−1^ (*u*_*c*_). The main contributor (about 90 %) to the combined uncertainty was the uncertainty of the correlation between time and mass diffused through the gel.

The elution efficiency *R* was 90.9 ± 1.6 % (*u*_*c*_). These values, together with their uncertainties, were applied for further calculations.

### pH working range

**Fig. 4:**
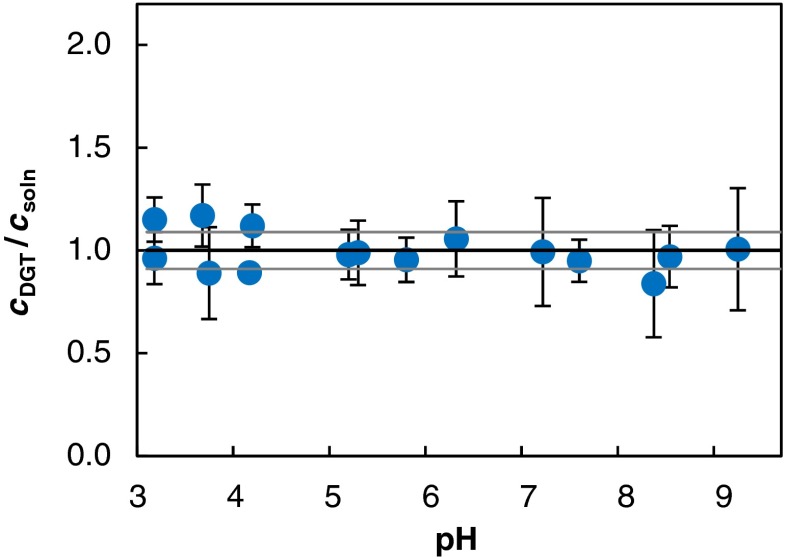
Determination of the pH working range. The *error bars* are *u*_*c*_, the 1.0 line is displayed with its uncertainty (*u*_*c*_ = 0.089)

The relative combined uncertainty of the *c*_DGT_/*c*_soln_ ratio was 8.9 %, and the major contributor to the uncertainty was the determination of individual *c*_DGT_ values. The relative combined uncertainty of an individual *c*_DGT_ value was on average 8 % (*u*_*c*_, *k* = 1). With respect to the combined uncertainty, the *c*_DGT_/*c*_soln_ was not significantly different from 1 for immersion solution pH values between 3 and 9 (Fig. 4). Slightly larger deviations of the mean *c*_DGT_/*c*_Soln_ values from 1 were observed in the pH range 3–5 compared to higher pH values.

### Gel capacity

**Fig. 5:**
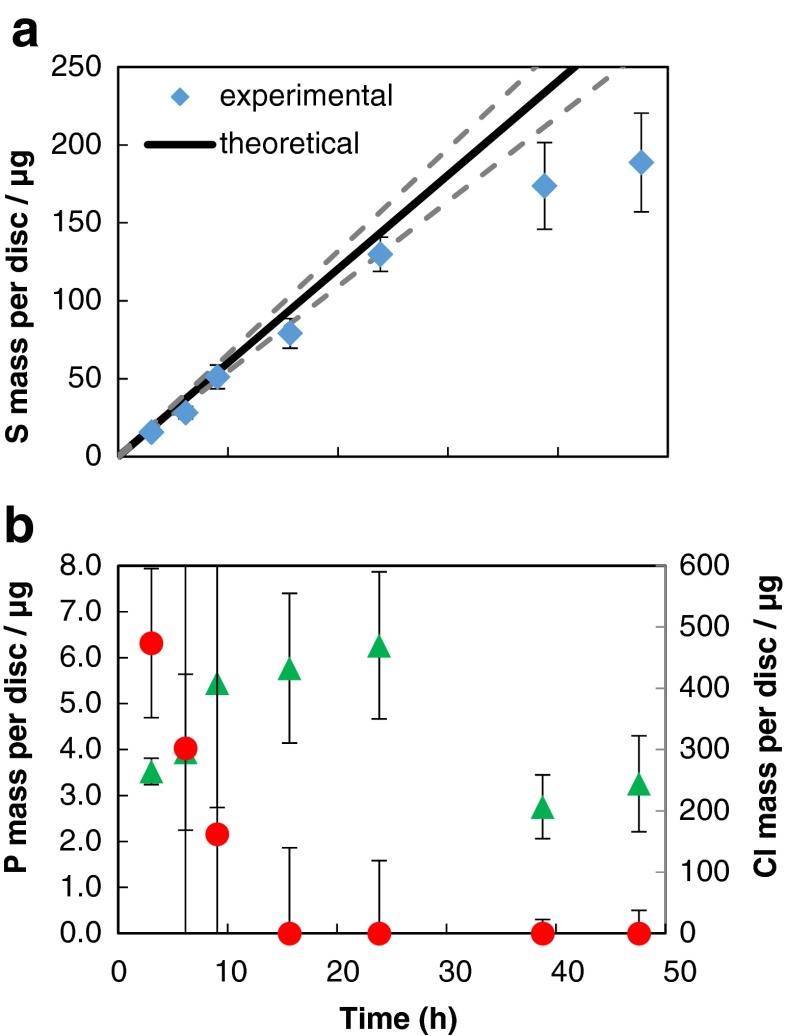
Sulfate uptake capacity. **a** Experimental (*blue diamonds*) and theoretical (black line ± 9 %, *u*_*c*_) sulfate uptake over time. **b** Release of chloride (*red points*) and phosphorus uptake (*green triangles*) by the gel over time. *Error bars* are *u*_*c*_

The relative combined uncertainty of the theoretical uptake line was 9.5 %, with the S measurement uncertainty being the main contributor (>75 %). The experimentally determined S uptake onto the gel after 24 h DGT deployment was up to 130 ± 11 μg S per disc and is in agreement with the theoretical value of 144 ± 13 μg S per disc (Fig. 5a). The concentration of Cl^−^ declined to <LOD (LOD, 10 μg L^−1^) after 15 h of DGT exposure, which was expected as the resin was used in its Cl^−^ form. The phosphate uptake reached its maximum after 24 h (6.3 ± 1.6 μg P per disc). At deployment times of 39 and 48 h, the P taken up by DGT declined to around half the maximum value (2.8 ± 0.7 μg per disc and 3.3 ± 1.0 μg per disc, respectively) (Fig. 5b).

The characteristics of the method are summarized in Table 2.

**Table 2:** DGT method characteristics

Parameter	Abbreviation	Value	Type of uncertainty	Unit
Method limit of detection	MDL	0.29	–	mg S L^−1^
Method blank resin gel loading	–	1.64 ± 0.63	SD (*n* = 4)	μg S per disc
Diffusion coefficient	*D*	9.83 × 10^−6^ ± 0.35 × 10^−6^	*u*_*c*_ (*k* = 1)	cm^2^ s^−1^
Elution efficiency	*R*	0.909 ± 0.016	*u*_*c*_ (*k* = 1)	–
Gel capacity	–	130 ± 11	SD (*n* = 3)	μg S per disc

### Comparison of DGT S with conventional soil S extraction techniques

**Fig. 6:**
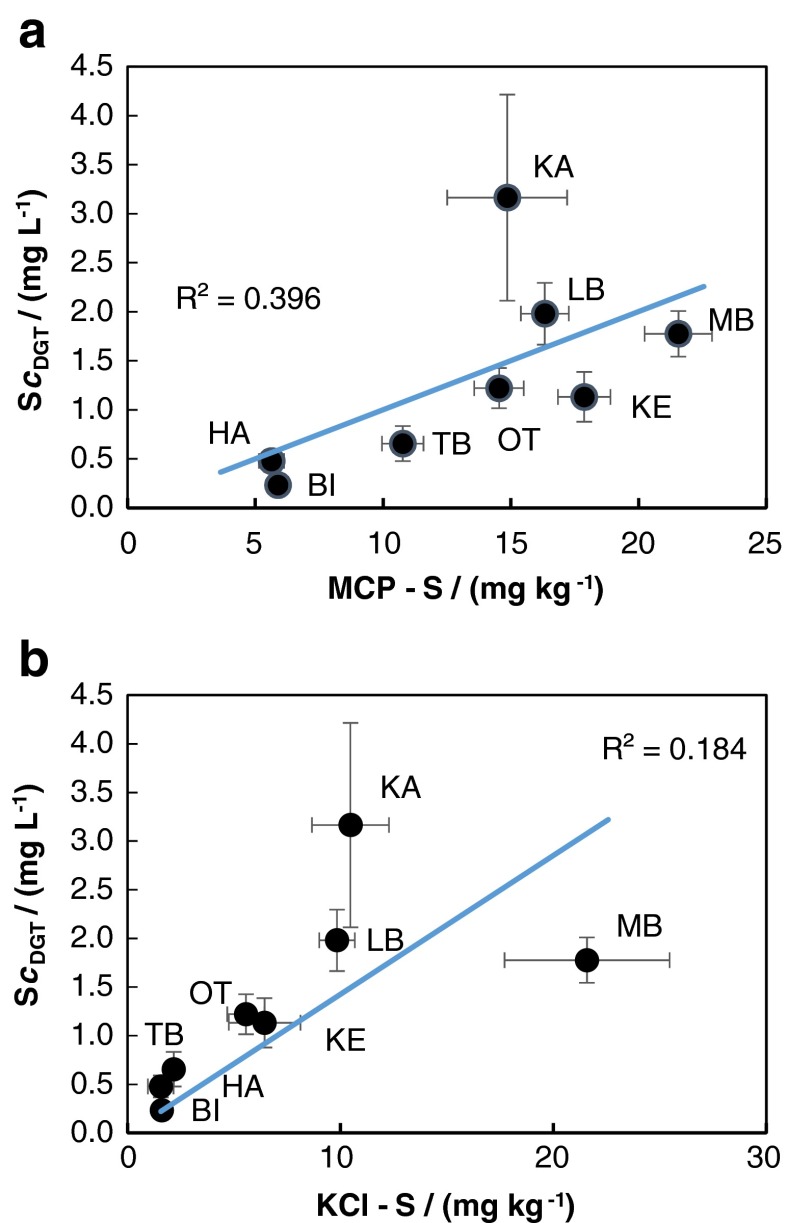
Correlation of DGT-S with S extracted by **a** MCP and **b** KCl-40. The line represents the linear regression line. *Error bars* are 1 SD

DGT S extracted from the experimental soils ranged between 1.5 and 20.2 μg, corresponding to *c*_DGT_ values of 0.23–3.16 mg L^−1^. The linear correlation coefficients of DGT-S with S extracted by the MCP and KCl-40 methods were low with *r* ^2^ = 0.40 (MCP) and *r* ^2^ = 0.18 (KCl-40) (Fig. 6).

## Discussion

The elevated background S levels on the unwashed protective membrane (Fig. 2) indicate the necessity to clean the membrane before use in order to prevent contamination of the sampler. No background S was detectable in the acid-washed membranes as well as in diffusive gel eluates. While S was determined in blank resin gel disc eluates, considerably more S was eluted from discs retrieved from non-deployed method blank DGT units. This indicates, that sampler assembly and handling may increase the background sulfate level on the resin gel, most likely due to sulfate being a very abundant chemical species even in a clean room laboratory setting. Monitoring of the sulfate background on method blanks is therefore a necessity for DGT sulfate analyses. However, the S masses accumulated on the resin gels in the soil experiment were 4–55 times higher than the blank S gel loading, indicating that this slightly elevated background S value is not a problem for soil sulfate testing using DGT.

The determined diffusion coefficient value (9.83 × 10^−6^ ± 0.35 × 10^−6^ cm^2^ s^−1^) was approximately 91.4 % of the SO_4_^2−^ *D*_25_ values for pure water (10.8 × 10^−6^ [25]) or seawater (10.7 × 10^−6^ [26]), which is expected as solute diffusion coefficients are generally lower in APA diffusive gels than in water [20, 27]. This is caused most likely by tortuous diffusion pathways through the acrylamide gel matrix. *D* was the same for all test solutions (1, 10, and 45 mg L^−1^ S) indicating that SO_4_^2−^ diffusivity is not concentration dependent, which is a pre-requirement for quantification of labile SO_4_^2−^ by DGT.

The elution efficiency (90.9 ± 1.6 %) showed that the sampled SO_4_^2−^ is not completely eluted from the resin gel. However, as the elution efficiency is almost constant (low combined uncertainty), a correction factor can be applied for the calculation of *c*_DGT_. Higher elution efficiency (~100 %) can be obtained if the sorbent in the resin gel is dissolved during elution, as is the case for ferrihydrite resin gels that are used for phosphate sampling by DGT [27], or when the resin gel is digested [18]. Application of an ion exchange resin in DGT leads to generally lower recoveries, e.g., when Chelex is used for cation sampling (70–82 % recovery [10]).

DGT can be expected to perform well for SO_4_^2−^ quantification in the pH range between 3 and 9. This wide pH range enables the application of the developed method to both arable and acidic (e.g., forest) soils. However, a larger uncertainty must be taken into account for the pH range 3–5.

The relative expanded uncertainty of an individual *c*_DGT_ value estimated in this study (typically 16 %, *U*_rel_ *k* = 2) was higher than the 10 % expanded uncertainty (*U*_rel_, *k* = 2) reported by Kreuzeder et al. [21]. The main contributor (up to 40 %) to the 8 % combined uncertainty was the uncertainty of the diffusion coefficient. The relative uncertainty of the *D*_25_ for SO_4_^2−^ in hydrogel (4 %) is twofold larger than the relative uncertainties of *D*_gel_ reported in [21].

The capacity of the gel was 130 μg S per gel disc, which equals 41 μg S cm^−2^ gel (calculated based on the sampling window area of 3.14 cm^2^) in the synthetic soil solution. As this value was obtained under high sulfate:anion ratios, the reported capacity can be considered a conservative lower-limit estimate. Based on the DGT soil analyses done in this study, and some of our ongoing work in which we obtained a maximum DGT S uptake of about 85 μg for 24 h deployments, this capacity is well suited for analyzing soil S using DGT.

In the capacity test, PO_4_^3−^ was continuously bound to the gel during the first 24 h. However, the mass of P measured later on declined to around half the maximum value. This behavior indicates that SO_4_^2−^ is taken up preferentially, as it replaced previously sorbed phosphate when the total gel loading was already high. The decrease of the Cl^−^ concentration in the eluates with time was expected as the chloride form of Amberlite IRA-400 was used and Cl^−^ was continuously replaced by other anions. The discrepancy between the sum of the bound SO_4_^2−^ and PO_4_^3−^ and the exchanged Cl^−^ can be explained by NO_3_^−^ bound on the resin. Although it can be assumed that NO_3_^−^ does not bind strongly to the resin and would be exchanged by SO_4_^2−^ present in the solution, its concentration in the synthetic soil solution was four times higher than the SO_4_^2−^ concentration. Part of the Cl^−^ was therefore probably exchanged for NO_3_^−^. It was not possible to confirm this assumption as 1 mol L^−1^ HNO_3_ was used as the eluting agent.

The developed method was successfully applied for a small set of selected soil samples. Sulfur sampled by DGT did not correlate well with that extracted by MCP and KCl-40. The low correlation coefficients between MCP and DGT (*r* ^2^ = 0.40) and KCl-40 and DGT (*r* ^2^ = 0.18) are likely linked to the differential sampling mechanisms of the methods applied. While extractions are based on a quasi-equilibrium between soil and extractant, DGT acts as an infinite sink technique that samples labile soil sulfate [13]. Visual inspection of Fig. 6 suggests that the two soils (KA, MB) might be outliers in the correlation. Excluding the soils KA and MB from the correlation between KCl-40 and DGT would increase the correlation coefficient to *r* ^2^ = 0.94. If the KA soil was excluded from the comparison of DGT with MCP, the correlation coefficient would increase to *r* ^2^ = 0.75. However, neither a statistical outlier test (Grubbs’ test, *p* > 0.05) identified KA and MB as outliers nor does the geochemical composition of the soil samples suggest a different behavior than the other soils. Clearly, a larger set of samples is needed to better understand the relation of the DGT and MCP/KCL-40 sampled sulfate fractions.

It has been shown that neither MCP nor KCl-40 corresponds well to the SO_4_^2−^ uptake of plants [15], while DGT has been shown to be a good predictor of plant-available nutrients and contaminants [28]. In an agronomical evaluation of the presented DGT method as a soil S test, Mason et al. [29] found that DGT predicted maize relative yield and S uptake better than the two extraction methods. Together with reports that DGT and plants utilize the same soil phosphate pools, while chemical batch extractions do not [12, 13], this indicates that DGT S uptake from soil resembles that of plants very well, while batch extraction methods are not efficient predictors of plant S uptake. Our study provides a first indication of the potential of DGT-S in soil testing; however, further investigation and validation of this approach is warranted.

## Conclusions

The presented DGT method showed great potential for soil SO_4_^2−^ sampling. The preference of the applied resin gel towards SO_4_^2−^ over other anions typically present in soil and soil solution, and the high capacity of the gel allow for SO_4_^2−^ sampling from soils of a pH range between 3 and 9.

As conventional extraction methods are not very representative of plant available soil S, the simple and quick DGT technique can deliver significant benefits. However, it has to be proved whether DGT samples the same soil S pool as plants. Direct comparison of plant and DGT uptake applying isotopic marking or analysis of stable S isotopes will enable this assumption to be tested.
